# Validity and reliability of the Korean version of the Digital Burnout Scale

**DOI:** 10.3389/fpubh.2024.1386394

**Published:** 2024-06-03

**Authors:** Seung-Yi Choi, Jung-Hee Kim

**Affiliations:** College of Nursing, The Catholic University of Korea, Seoul, Republic of Korea

**Keywords:** digital burnout, digital aging, digital deprivation, emotional exhaustion, university students

## Abstract

**Objectives:**

To investigate the factor structure and verify the validity and reliability of the Korean version of the Digital Burnout Scale (DBS) among Generation Z university students.

**Methodology:**

The World Health Organization guidelines were employed in the forward and back translation, synthesis, cross-cultural adaptation, and pre-survey phases to result in the Korean version of the DBS. The Korean version was then used to collect data from 330 university students online. Construct, convergent, discriminant, and concurrent validity, and internal consistency were assessed.

**Findings:**

The Korean version of the DBS had three subscales (digital aging, digital deprivation, and emotional exhaustion) and included 24 items. The results of the confirmatory factor analysis indicated adequate model fit indices. Convergent, discriminant, and concurrent validity were satisfactory. The Cronbach’s ⍺ for the overall scale was 0.95.

**Conclusion:**

The Korean version of the DBS has good reliability and validity and can be used as a tool to assess the level of risk of digital burnout and provide appropriate support and intervention.

**Implications:**

The Korean version of the DBS will serve as a framework for developing healthy digital education by grasping individual characteristics. Longitudinal research is recommended to identify factors that cause digital usage and burnout for various age groups.

## 1 Introduction

The development and widespread use of information and communication technologies have normalized the everyday use of various forms and functions of digital devices in all aspects of daily life (1, 2). The Coronavirus Disease 2019 (COVID-19) pandemic has been a catalyst for the digital transformation of daily life, changing traditional face-to-face interactions to non-face-to-face approaches through the application of digital technology (3). Consequently, many aspects of daily life depend on digital technology (4). The ease and speed with which digital technology can be used affects the quality of life; however, the digitization of daily life has various effects due to excessive time spent on digital devices, such as mental and behavioral health issues, academic performance problems, sleep issues, and musculoskeletal health problems (1, 5, 6).

The current generation of university students, Generation Z, is referred to as “Digital Natives” (7, 8). Born between the mid-1990s and early 2000s, they have grown up surrounded by digital devices since birth (9, 10). Having never experienced life before the Internet, they are sensitive to and active users of digital technology, with the smartphone being the most commonly used device regardless of gender (7). They prefer digital media for receiving diverse types of information and favor communication through digital technology over direct contact with people (11). Since there are various ways to express oneself online and form relationships, individuals can enjoy more freedom in interacting with others by building an image that differs from reality (12). Consequently, people become attendants who present an edited version of themselves that they believe others will best accept and often manage other people’s perceptions about them through what they post (13). Furthermore, online self-expression can differ from offline self-expression (14). This difference is confusing and may create a conflict between one’s online image and who one really is (15).

It is estimated that Generation Z individuals watch their smartphone screens for an average of 4–5 h a day (11). This generation has grown up with digital technology, is familiar with it, is greatly influenced by it, and has a strong presence on various digital platforms (16). They use it for various tasks such as checking emails, finding directions, taking pictures, buying, and entertainment (10). Of the Generation Z population, 98% have a smartphone and 97% have a social media account (17). However, problematic digital use can lead to feelings of isolation, which can lead to other chronic psychological disorders (9). Digital devices can affect the social, physical, and psychological functions of Generation Z and can cause neurological impairments including emotional, mental, and physical health problems such as insomnia, irritability, depression, social isolation, and musculoskeletal problems (8, 9, 11, 16, 17).

The excessive use of digital devices negatively impacts the academic performance, achievement, and learning adaptation of university students (2). Using digital devices until bedtime can lead to insomnia, decreased concentration, and less participation in academic activities, resulting in lower academic performance (18, 19). Therefore, the use of digital devices could disrupt learning and reduce motivation to learn (20). In addition, the improper and excessive use of digital education and learning technologies may lead to academic exhaustion (21). Excessive use of smartphones for non-academic purposes can hinder concentration on studies and lead to procrastination in completing assignments (21, 22). Several studies found a correlation between academic exhaustion and mobile phone use (19–24). Academic burnout is a common issue today, given that most learning takes place on computers and the boundary between school and the online world is blurred.

Academic burnout originates from the concept of job burnout and is usually characterized by three dimensions: emotional exhaustion, cynicism, and reduced academic efficacy (21, 25, 26). In the academic context, these dimensions translate to feeling exhausted because of study demands, having a cynical and detached attitude toward one’s studies, and feeling incompetent as a student (26, 27). Academic burnout can overwhelm students and lead to a loss of interest in activities they once enjoyed (19, 21, 24). Students experiencing academic exhaustion tend to lack self-control and use digital devices excessively (21, 24). Furthermore, phenomena such as nomophobia (fear of being without a mobile phone) (5), sleep disorders (18, 19), eye dryness (28), musculoskeletal health problems (29), reduced activity (30), and obesity may occur when smartphones are not available (1). Therefore, it is imperative to promptly assess and implement preventive measures to effectively address these issues.

Erten and Özdemir (31) developed the Digital Burnout Scale (DBS) in English and Turkish to measure the extent of digital burnout resulting from the use of digital devices. This scale facilitates proper assessment of and finding solutions to health issues. Even in the post-COVID-19 era, the digitalization of daily life has accelerated with the expansion of non-face-to-face and online stores, diversification of EdTech learning contents, and growth of the remote work collaboration tools market (16, 32, 33). Recognizing the increased risk of health issues related to digital exposure and overuse as daily patterns change owing to digitization, systematic prevention and management measures are needed (31). As such, using tools that can appropriately assess the physical, mental, and psychological issues of an individual is crucial (31).

During the COVID-19 pandemic, the concept of digital burnout emerged owing to the use of complementary and accessible technologies (31, 33–36). In our daily lives, we are constantly exposed to various digital devices, and without realizing it, may experience digital burnout from spending too much time on these (31, 34). However, individuals may be unaware of their situation, which makes it challenging to find solutions (3). Thus, the concept of digital burnout, a comprehensive psychological and physical problem caused by excessive exposure to digital culture, should be measured and evaluated. Therefore, this study aims to find individual levels of digital burnout in the digitalization era to enable careful observation and awareness of one’s digital usage patterns. Furthermore, it aims to evaluate the Korean version of the DBS through rigorous tool translation and validity and reliability surveys.

The next section of this paper provides a review of the current literature. Then, the methodology and findings of the study are presented, followed by the discussion, theoretical and managerial implications, and conclusion. Finally, the limitations of the study are delineated.

## 2 Literature review

Burnout syndrome is an individual response to chronic work stress that develops progressively and can eventually become chronic and negatively impact the individual’s health (33, 37). Maslach (38) introduced and defined the concept of burnout as a gradual process of fatigue, cynicism, and reduced commitment among social care professionals. After several empirical studies, Maslach in collaborated with other authors (39, 40) reformulated the concept and elaborated a more rigorous and operational definition of burnout as a psychological syndrome characterized by emotional exhaustion, cynicism or depersonalization, and a reduced sense of professional efficacy (41). Burnout is a syndrome characterized by long-term physical and emotional fatigue and low work performance, which can lead to unproductive, inactive, and negative attitudes toward work (15, 34). Burnout negatively affects quality of life as it decreases mental, physical, and emotional health (42). Some signs and symptoms of digitally induced burnout are similar to those of normal burnout. The main difference between the two is evident in the long-term use of digital devices to the extent that individuals cannot be completely separated from their digital lives even during breaks (31, 33). Digital burnout is characterized by sleep deprivation and reduced efficiency at work, family issues, fatigue, stress, loss of interest, depersonalization, difficulty managing emotions, and physical and mental problems (3, 6, 31, 33, 34). Since burnout is both physical and psychological, people may experience different forms of burnout in their lives (31).

Erten and Özdemir (31) refined the concept of digital burnout by identifying three characteristics: digital aging, digital deprivation, and emotional exhaustion. Digital aging is one’s inability to strike a balance between the real world and the virtual world as a result of spending excessive time on digital platforms. Digital deprivation refers to the state in which one feels bad, physically or psychologically, when staying away from digital platforms. Emotional exhaustion refers to the depletion or draining of emotional resources (31, 34).

Excessive time spent online incurs a major risk of digital addiction and promotes digital exhaustion (43). Digital technologies lead individuals to experience a “fear of missing out,” and the anxiety it provokes is similar to that experienced with other addictions (33, 44). When this digital dependency is normalized and accepted, individuals experience a reduced ability to carry out activities that encourage them to disconnect from their digital devices (33). Consequently, digital burnout may occur (31). During these instances, individuals may experience a range of emotions and exhibit corresponding behaviors including tension, anxiety, dread, feelings of inadequacy, a sense of constriction, and difficulties concentrating (6). Therefore, it can be useful to consider digital addiction when analyzing digital burnout. This approach will help better understand and manage health and well-being in a digital environment (33).

Although digital tools offer practicality and convenience, their excessive use can act as a double-edged sword, contributing to digital burnout (3, 43). This phenomenon, as explained by demands–resources theory, suggests that burnout originates from an imbalance between work demands and available resources, with insufficient recovery exacerbating physical and mental exhaustion (41). When demands surpass resources, fatigue ensues, potentially leading to chronic fatigue and eventual burnout (41).

As the time spent using digital devices in daily life rapidly increases, university students find themselves continuously exposed to and utilizing these devices for their studies (16, 21, 45). While such use of digital devices can revolutionize the academic environment and enhance efficiency (22), it also heightens the risk of digital burnout (3, 34). Through online lectures, learning management systems, and online materials, students can easily access course materials, but this ease of access can also hinder their ability to concentrate on their studies (21). Factors such as technical issues and education quality from online lectures can cause stress among students regarding their academic performance (21). Additionally, students may allocate a significant amount of time to activities such as social networking, online video watching, and gaming (10, 11), which can reduce the time and energy they have available for their studies, ultimately leading to digital burnout (3, 34).

In conclusion, the continuous use of and exposure to digital devices in both academic and daily life settings can elevate the risk of digital burnout among university students (3). As such, the purpose of this study is to confirm the negative effects of digital use on individuals, including various internal and external stimuli, as well as emotional and cognitive overload. Specifically, we aim to evaluate the impact of digitalization on burnout and, consequently, the digital burnout experienced by Generation Z college students.

However, evaluations conducted in a specific context without considering the suitability of the measurement tools within that context may compromise the validity of the measurement and research results (28). The classical method for verifying a scale is factor analysis (46), and the DBS currently in use is based on the results of such an analysis confirming its validity among a population of 707 participants in Turkey (31). However, it needs to be determined whether the proposed factor structure in the initial population remains consistent among Generation Z university students in Korea to determine whether the DBS is a valid tool for measuring their level of digital burnout. Thus, the purpose of this study is to investigate the factor structure and to verify the validity and reliability of the Korean version of the DBS among Generation Z university students. Research on the validity of the Korean version of the DBS will contribute to establishing the concept of digital burnout and fostering a healthy digital culture in Korea.

## 3 Methods

### 3.1 Study design

This study was a methodological study with a cross-sectional design to investigate the factor structure in Korean Generation Z university students and verify the validity and reliability of the DBS. A cross-sectional design is an appropriate method for determining the prevalence of a disease, attribute, or phenomenon in a study sample, enabling investigators to describe their study sample and review associations between collected variables (47). It is effective to analyze each item when verifying the appropriateness of a tool, as the item is the most basic unit constituting that tool (48–50).

### 3.2 Participants

The participants in this study were first-, second-, third-, and fourth-year undergraduate students who resided in Korea, as well as individuals who had experience using digital devices (smartphones, tablets, wearables, etc.). Participants belonged to Generation Z, meaning they were born in or after 1995 (9, 10). Participants residing abroad or foreign nationals attending the universities were excluded. The survey was conducted online. A questionnaire was posted on various online communities frequently visited by Korean students to prepare for exams, find jobs, and learn languages. Detailed information on the study was provided on the first screen of the online survey. Subjects could only participate in the survey if they voluntarily agreed to do so. Filtering was set so that people who did not meet the inclusion criteria could not respond. Anonymity and confidentiality of the data were maintained to protect the rights of the research participants. Data were collected between July 26 and August 2, 2023, and participants were allowed to withdraw from the study at any time. All participants who completed the survey were given a small gift in return. To determine the validity of the tool, a sample size of at least five or 10 times the number of items is required (46). Since the original tool consisted of 24 items, a sample size of 240 was considered appropriate. However, considering that a minimum of 300 participants is suitable for confirmatory factor analysis (CFA) (51), and accounting for a 10% incomplete response rate, the required sample size was set at 330 participants. We collected 330 questionnaires, and the analysis was performed using the final dataset of 330 participants with no missing data.

### 3.3 Instruments

#### 3.3.1 Digital Burnout Scale

The DBS was developed to measure burnout resulting from the use of digital devices (31). It consists of 24 items across 3 subscales, including content related to digital aging (12 items), digital deprivation (6 items), and emotional exhaustion (6 items). Each item in the DBS is measured on a 5-point Likert scale ranging from “strongly disagree” (1 point) to “entirely agree” (5 points). The total score ranges from 24 to 120, with high scores indicating high levels of digital burnout. During its development, this tool was validated with 286 high school students, 288 university students, and 133 graduates in Turkey, and its reliability was established based on Cronbach’s *α* = 0.94 (31). In this study, Cronbach’s *α* = 0.96.

#### 3.3.2 Korean version of the Digital Addiction Scale

The Korean version of the Digital Addiction Scale (K-DAS), developed by Kesici and Tunç (52) and adapted for Korean by Kim et al. (53), was used to verify concurrent validity. The K-DAS consists of 19 items measuring compulsive use (8 items) and the negative consequences thereof (11 items). Each item is scored on a 5-point Likert scale, with a total score ranging from 19 to 95. A higher total score indicates a higher level of digital addiction. In this study, the reliability was Cronbach’s *α* = 0.94.

#### 3.3.3 Korean version of the Maslach Burnout Inventory-Student Survey

The Korean version of the Maslach Burnout Inventory-Student Survey (MBI-SS), developed by Schaufeli et al. (27) and adapted for Korean by Lee and Lee (54), was used to verify concurrent validity. The Korean version of the MBI-SS consists of 14 items related to exhaustion (5 items), inefficacy (5 items, reverse-coded), and cynicism (4 items). Each item is rated on a 7-point Likert scale, with a total score ranging from 14 to 98. A higher total score indicates a higher level of academic burnout. In this study, the reliability was confirmed with Cronbach’s *α* = 0.87.

### 3.4 Procedures

#### 3.4.1 Translation

The Korean version of the digital burnout measurement tool was translated according to the World Health Organization guidelines (55). In the primary translation stage, two professional translators proficient in both English and Korean independently translated the measurement items from the original tool, which was written in English. Thereafter, in the expert panel stage, two nursing professors with experience in tool development and proficiency in both Korean and English reviewed the tool translated in the primary stage. During this stage, they confirmed and completed the draft items in Korean, ensured the translated items accurately represented the measurement concepts and had appropriate expressions, and addressed any items that required modification owing to cultural differences. Afterward, in the back-translation stage, a bilingual English-speaking professor was commissioned to translate the reviewed version. The questionnaire, completed through translation and back-translation, was constituted as a preliminary tool through discussions among a linguistics professor with experience in tool development, two nursing professors, and the back-translator. They confirmed and revised the discrepancies between the back-translated and original tools, expressions affected by cultural differences, and any distortions of meaning. Finally, after receiving grammatical corrections from a linguistics scholar specializing in Korean that did not alter the meaning, the Korean version of the preliminary tool was confirmed.

#### 3.4.2 Preliminary survey

For the preliminary investigation, samples were selected using the same criteria as for the main investigation. Ten university students participated voluntarily and were instructed to complete a self-report questionnaire. The preliminary investigation aimed to identify confusing expressions or difficult questions, and measure response time. The results of the preliminary investigation indicated that the response time was approximately 7–10 min and that no expressions or questions posed difficulties for the respondents. Consequently, the final tool was completed without any modifications to the questions.

### 3.5 Ethical considerations

This study was conducted after obtaining approval from the Institutional Review Board (IRB No. MC23QASI0061) of the institution with which the researchers are affiliated to protect and respect the rights of research participants.

### 3.6 Data analysis

Data were analyzed using IBM SPSS Statistics 22 and Amos 18 software (IBM Corp., Armonk, NY, United States). IBM SPSS Amos is a powerful structural equation modeling software that enables researchers to support their studies and hypotheses by extending standard multivariate analysis methods including regression, factor analysis, correlation, and an analysis of variance. With SPSS Amos, one can build attitudinal and behavioral models that reflect complex relationships more accurately than with standard multivariate statistics techniques using either an intuitive graphical or programmatic user interface (56).

The CFA was conducted to assess construct validity, and principal component factor analysis using the Varimax method was employed for parameter estimation. Model fit was evaluated based on various fit indices including the absolute fit indices (*χ*^2^), *χ*^2^/degree of freedom, root mean square error of approximation (RMSEA), standardized root mean residual (SRMR), comparative fit index (CFI), and Tucker–Lewis index (TLI) (46, 56). As *χ*^2^ can be influenced by sample size or model complexity, fit indices other than *χ*^2^ (*p* > 0.05), including *χ*^2^/df ≤ 3, RMSEA ≤0.08, SRMR ≤0.08, CFI ≥ 0.90 and TLI ≥ 0.90, were considered to assess overall fit (46, 57–59).

Convergent and discriminant validity were assessed using standardized estimates (β), construct reliability, average variance extracted (AVE), and inter-factor correlation coefficients (46). Convergent validity of the items was confirmed through standardized factor loadings (*β*) ≥ 0.50, AVE ≥ 0.50, and CR ≥ 0.70. Discriminant validity was assessed based on the criterion of not including 1 within the range of *r* ± 2 × Standard Error (46). The normality of each item was evaluated based on absolute skewness values of less than 3 and absolute kurtosis values of less than 10 (57). Concurrent validity was assessed by assuming significant positive correlations between the Korean versions of the DBS and K-DAS, and between the Korean versions of the DBS and MBI-SS. Pearson’s correlation analysis was performed for this purpose. Finally, internal consistency reliability was verified using Cronbach’s *α*.

## 4 Findings

### 4.1 Participants’ characteristics

The mean age of participants was 21.27 ± 1.70 years, with 42.7% males and 57.3% females. The majority were second-year students (45.8%) and humanities majors (30.3%). The average daily usage of digital devices was 5.88 ± 2.54 h, and academic grade was predominantly categorized as “Middle” (69.4%), “Low” (17.0%), and “High” (13.6%). Devices were primarily used for social network services (SNS; 39.1%), instant messenger (26.7%), and video-sharing platforms (20.9%) (Table 1).

**Table 1:** Characteristics of participants (*N* = 330).

Characteristic	Categories	*n* (%) or *M* ± *SD*
Gender	Male	141 (42.7)
	Female	189 (57.3)
Age (years)		21.27 ± 1.70
Grade	1st	84 (25.5)
	2nd	151 (45.8)
	3rd	60 (18.2)
	4th	35 (10.6)
Major	Humanities	100 (30.3)
	Social Science	61 (18.5)
	Natural Science	41 (12.4)
	Engineering	41 (12.4)
	Arts and Sports	18 (5.5)
	Medicine	63 (19.1)
	Education	6 (1.8)
Residence	Seoul	131 (39.7)
	Gyeonggi/Incheon	91 (27.6)
	Gangwon	14 (4.2)
	Chungcheong	28 (8.5)
	Jeolla	38 (11.5)
	Gyeongsang	27 (8.2)
	Jeju	1 (0.3)
Digital device day use time (hrs.)		5.88 ± 2.54
Academic grade	High	45 (13.6)
	Middle	229 (69.4)
	Low	56 (17.0)
Digital device use purpose	Social Network Service	129 (39.1)
	Video-Sharing Platform	69 (20.9)
	Instant Messenger	88 (26.7)
	Games	25 (7.6)
	Education Platform	13 (3.9)
	Investment Platform	6 (1.8)

M, mean; SD, standard deviation.

### 4.2 Item analysis

The mean score for each item ranged from 2.27 ± 1.24 to 3.36 ± 1.13. The skewness for each item ranged from −0.55 to 0.75, and kurtosis ranged from −1.16 to −0.40, which met the criteria. The correlation coefficients between each item and the overall items ranged from 0.54 to 0.81, indicating that each item contributed significantly to its respective subscale (Table 2).

**Table 2:** Reliability of the Korean version of the DBS (*N* = 330).

Factor	Item no.	Item description	*M* ± *SD*	Skewness	Kurtosis	ITC	Cronbach’s *α*
Digital Aging	1	I have an attention deficit.	2.77 ± 1.24	0.13	−1.00	0.74	0.939
	2	I think that I will lose my mind 1 day.	2.31 ± 1.24	0.75	−0.43	0.73	
	3	I sometimes feel like my mind gets blurred.	2.51 ± 1.32	0.37	−1.16	0.72	
	4	I feel stressed.	3.36 ± 1.13	−0.55	−0.40	0.60	
	5	Either my hand or my body aches as a result of constantly typing and checking messages.	2.42 ± 1.21	0.47	−0.84	0.76	
	6	I started thinking that I have symptoms of depression.	2.47 ± 1.30	0.48	−0.94	0.71	
	7	A feeling of loneliness dominates me.	2.52 ± 1.31	0.42	−1.03	0.78	
	8	I am confused about my status.	2.51 ± 1.30	0.41	−0.97	0.76	
	9	I feel restricted.	2.50 ± 1.28	0.39	−1.05	0.74	
	10	I cannot establish a balance between the real and virtual worlds.	2.27 ± 1.24	0.59	−0.86	0.77	
	11	I spend long periods of time in the virtual world with digital devices.	2.76 ± 1.27	0.08	−1.10	0.68	
	12	I speak and look around less.	2.69 ± 1.24	0.18	−1.00	0.75	
Digital Deprivation	13	I feel uneasy when I do not have an Internet connection or am offline.	2.96 ± 1.26	−0.06	−1.13	0.66	0.884
	14	I always think about the message I just received and what is happening.	2.93 ± 1.21	−0.05	−1.02	0.60	
	15	I feel naked when I do not have my digital devices (phone, tablet, computer, etc.) with me.	2.98 ± 1.23	−0.14	−1.04	0.64	
	16	I check my tweets, Facebook account, emails, and messages all the time. If I do not, I feel weird or anxious.	2.92 ± 1.23	−0.07	−1.07	0.54	
	17	I feel powerless when I do not have an Internet connection or am offline.	3.02 ± 1.24	−0.12	−1.07	0.67	
	18	I am most afraid of losing or forgetting my phone. This thought bothers me.	3.17 ± 1.31	−0.28	−1.07	0.59	
Emotional exhaustion	19	I feel exhausted because of the virtual and digital worlds.	2.56 ± 1.24	0.30	−1.01	0.76	0.889
	20	I almost feel nothing about events and situations around me.	2.41 ± 1.21	0.44	−0.90	0.74	
	21	I have become intolerant of and desensitized to the people around me.	2.64 ± 1.25	0.33	−0.97	0.81	
	22	I have become impatient.	2.73 ± 1.29	0.25	−1.02	0.77	
	23	I have become quick-tempered.	2.86 ± 1.28	0.03	−1.13	0.71	
	24	I think that my relationships and communications with people have been weakened.	2.80 ± 1.28	0.16	−1.04	0.76	

DBS, Digital Burnout Scale; ITC, item-total correlation; M, mean; SD, standard deviation.

### 4.3 Construct validity

#### 4.3.1 Confirmatory factor analysis

**Figure 1:**
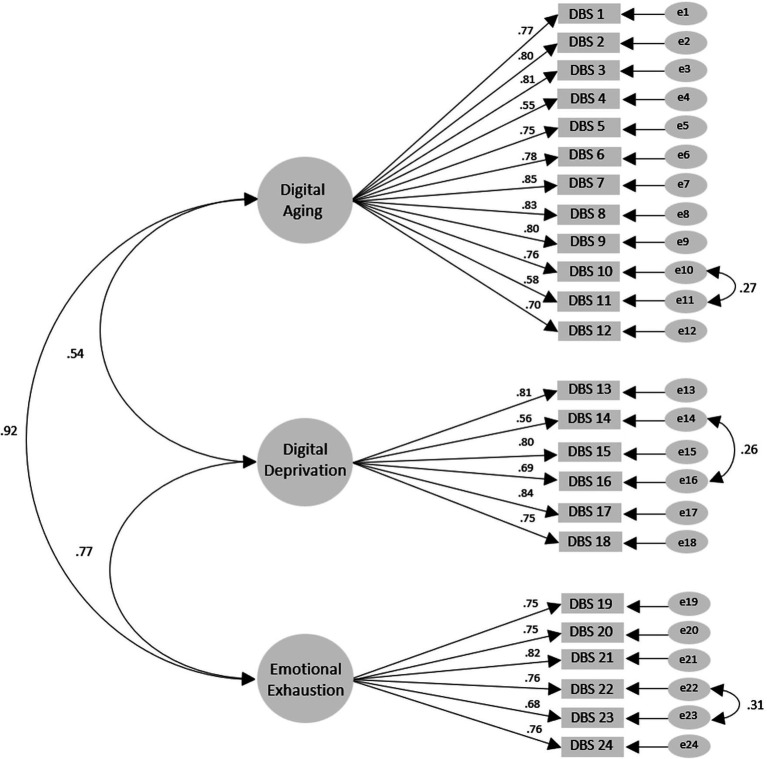
Measurement model for the Korean version of the DBS.

The CFA results showed that *χ*^2^ = 804.94 (df = 249, *p* > 0.001), *χ*^2^/df = 3.23, RMSEA = 0.08, SRMR = 0.07, TLI = 0.89, and CFI = 0.90, indicating that some fit indices did not meet the criteria. Thus, adjustments were made to the model by sequentially using item paths within factors, starting with items with modification indices greater than 10, to avoid influencing the relationships between factors [(46, 58); Figure 1]. The adjusted model fit, excluding the sample size-sensitive *χ*^2^ = 732.53 (df = 246, *p* > 0.001), met the criteria with *χ*^2^/df = 2.98, RMSEA = 0.08, SRMR = 0.07, TLI = 0.90, and CFI = 0.91 (Table 3). The fit indices provided evidence confirming the three-factor Korean version of the DBS model.

**Table 3:** Factor structure of the Korean version of the DBS using confirmatory factor analysis (*N* = 330).

Factor	Item no.	Estimate		*SE*	C.R. (*p*)	*r* (*r*2) (*r* ± 2 × *SE*)			AVE	CR
		*B*	*β*			Digital aging	Digital deprivation	Emotional exhaustion		
Digital aging	1	1.00	0.77	–	–	1			0.57	0.94
	2	1.02	0.79	0.065	15.65 (<0.001)					
	3	1.11	0.81	0.069	15.98 (<0.001)					
	4	0.66	0.56	0.063	10.45 (<0.001)					
	5	0.96	0.76	0.065	14.75 (<0.001)					
	6	1.04	0.77	0.069	15.04 (<0.001)					
	7	1.16	0.85	0.068	16.99 (<0.001)					
	8	1.12	0.83	0.068	16.52 (<0.001)					
	9	1.07	0.80	0.067	15.88 (<0.001)					
	10	0.99	0.76	0.066	14.97 (<0.001)					
	11	0.79	0.60	0.070	11.26 (<0.001)					
	12	0.91	0.70	0.067	13.52 (<0.001)					
Digital deprivation	13	1.00	0.81	–	–	0.55^**^ (0.30) (−0.11, 0.19)	1		0.56	0.88
	14	0.66	0.56	0.064	10.34 (<0.001)					
	15	0.97	0.80	0.060	16.06 (<0.001)					
	16	0.83	0.69	0.063	13.20 (<0.001)					
	17	1.02	0.84	0.060	17.11 (<0.001)					
	18	0.97	0.75	0.065	14.81 (<0.001)					
Emotional exhaustion	19	1.00	0.77	–	–	0.85^**^ (0.72) (−0.10, 0.24)	0.70^**^ (0.49) (−0.11, 0.24)	1	0.58	0.89
	20	0.96	0.75	0.067	14.37 (<0.001)					
	21	1.09	0.83	0.068	16.07 (<0.001)					
	22	1.05	0.77	0.078	13.47 (<0.001)					
	23	0.91	0.68	0.072	12.66 (<0.001)					
	24	1.02	0.76	0.070	14.47 (<0.001)					
Fitness index	*χ*^2^ (*p*-value)				*χ*^2^/df	RMSEA	SRMR	TLI	CFI	
Model	732.53 (*p* < 0.001)				2.98	0.08	0.07	0.90	0.91	

AVE, average variance extracted; C.R., critical ratio; CR, construct reliability; CFI, comparative fit index; DBS, Digital Burnout Scale; df, degree of freedom; r, correlation coefficient; RMSEA, root mean square error of approximation; SE, standard error.; SRMR, standardized root mean residual; TLI, Tucker–Lewis index; β, standardized estimate. ^**^p < 0.001.

#### 4.3.2 Convergent and discriminant validity

The results for convergent validity showed that the *β* values for all items ranged from 0.56 to 0.85. The AVE ranged from 0.56 to 0.58, and the CR from 0.88 to 0.94, satisfying all three criteria to verify convergent validity. Discriminant validity was confirmed as the range of *r* ± 2 × Standard Error was from 0.10 to 0.24, not including 1, which aligns with the criteria (Table 3).

#### 4.3.3 Concurrent validity

Concurrent validity was analyzed in relation to digital addiction and academic burnout. Digital burnout showed significant positive correlations with digital addiction (*r* = 0.73, *p* < 0.001) and academic burnout (*r* = 0.66, *p* < 0.001), indicating concurrent validity (Table 4).

**Table 4:** Concurrent validity with the Korean version of the DBS (*N* = 330).

Scales	K-DAS	Korean version of MBI-SS
	*r* (*p*)	
Korean version of the DBS	0.73 (<0.001)	0.66 (<0.001)
Digital aging	0.58 (<0.001)	0.62 (<0.001)
Digital deprivation	0.76 (<0.001)	0.50 (<0.001)
Emotional exhaustion	0.72 (<0.001)	0.64 (<0.001)

DBS, Digital Burnout Scale; K-DAS, Korean version of the Digital Addiction Scale; MBI-SS, Maslach Burnout Inventory-Student Survey.

### 4.4 Internal consistency reliability

The Cronbach’s *α* for the Korean version of the DBS was 0.95. The internal consistency range for the three factors was between 0.88 and 0.94 (Table 2).

## 5 Discussion

The purpose of this study was to investigate the factor structure of Korean Generation Z university students and to verify the validity and reliability of the DBS. The three-factor measurement model of the Korean DBS was deemed appropriate. These results are consistent with the measurement model developed by Erten and Özdemir (31) for Turkish high school students, university students, and graduates, indicating similarity with the original model.

The first factor, “digital aging,” consists of 12 items. Digital aging refers to the imbalance between the real and virtual worlds caused by excessive time spent on digital platforms (31). When digital activities negatively affect individuals’ abilities and energy, they induce mental and physical fatigue, resembling the aging process (3). Participants in this study spent 5.88 ± 2.54 h per day using digital devices. Increased exposure to digital devices and content may result in adverse non-specific symptoms and physiological issues. Previous studies indicated that using digital devices for >2 h can increase eye fatigue and lead to dry eye syndrome (28, 60).

The use of digital devices often involves sitting in a forward-leaning posture, which leads to constant changes in posture and muscle activity (29). This increases the mechanical load on the spine, causing muscle fatigue and potentially resulting in neck and back pain (29). In previous studies investigating musculoskeletal pain among university students, the use of digital devices was associated with frequent pain in the neck, shoulders, lower back, wrists, and hands (29, 61). Prolonged use of digital devices has also been linked to a reduction in overall physical activity (30). Several studies have indicated an elevated risk of obesity and higher body mass index among individuals who spend considerable time on computers or watching television (62, 63). In addition, using smartphones has been associated with decreased walking speed and impaired balance (64). A causal link has also been found between mental health symptoms and issues stemming from excessive digital use (33, 45). For this, studies have emphasized the elevated risk of emotional vulnerabilities associated with excessive digital device use, such as heightened depression, diminished self-esteem, and increased stress perception (3, 31). Consequently, prolonged and excessive use of digital devices can contribute to physical problems, encompassing musculoskeletal and ocular issues, as mentioned, regardless of age. The symptoms related to digital aging may accrue over time, underscoring the importance of prevention and early intervention.

The second factor, “digital deprivation,” consists of six items that measure how negatively individuals feel physically and psychologically when they distance themselves from digital platforms. In this regard, on a 5-point scale, the statement scoring the highest (3.17 points) was: “I am most afraid of losing or forgetting my phone. This thought bothers me.” This indicates that the high portability of digital devices can lead to obsessive use, resulting in negative outcomes (31, 53). This corresponds to nomophobia (5, 7), where individuals feel fear without their smartphone, emphasizing the contextual aspect.

Generation Z students have incorporated communication through social media and online platforms into their daily routines; it has become a natural habit that requires little conscious effort (10, 11). Their main use of digital platforms, such as SNS, revolves around communicating with friends, sustaining connections, or gaining additional information. However, the lack of digital devices can evoke feelings of anxiety and loss if communication via digital platforms is interrupted (9). Generation Z students receive education in a learning environment that utilizes digital technologies such as videos, simulations, online quizzes, case studies, and social media (16). Therefore, the effect of digital deprivation is presumed to be pronounced when they do not have access to digital devices. Given the overall digitization of living and learning environments, in which digital device usage is prevalent (6), appropriate use of digital devices provides convenience and familiarity in daily life. However, excessive usage can lead to mental and physical health problems (3, 31, 33, 34). Therefore, understanding an individual’s digital usage patterns and quantity is crucial. Furthermore, considering the potential decrease in real-world communication and the risk of social weakening and isolation associated with increased activity in the digital world (13, 14), it is essential to assess digital deprivation due to individual digital usage and to implement measures to minimize the negative impact on social relationships.

The third factor, “emotional exhaustion,” comprises six items measuring emotional fatigue resulting from the use of digital devices. Efforts to learn and apply new technologies can induce stress and lead to emotional exhaustion (65). Emotional exhaustion is a contemporary adaptation disorder resulting from an unhealthy coping mechanism involving the use of new technologies, often referred to as “Technostress” (45). It involves a broad spectrum of emotional stress experienced through using information systems (65). As students widely use mobile technology, social media, and various educational technologies (e.g., e-learning systems, online resources, PowerPoint slides, and podcasts), some are prone to experiencing high levels of technostress (21, 45). Consequently, the diverse use of digital devices can trigger emotional exhaustion, thereby depleting emotional resources and causing mental and physical fatigue. This depletion may result in overload, inefficient information processing, confusion, loss of control, psychological stress, increased depressive symptoms, and other personality changes and emotional issues (3, 6, 33). The interaction between emotional exhaustion and the use of digital devices is complex and can vary based on user characteristics, usage patterns, living environments, and other factors (31, 34).

The concurrent validity of the Korean version of the DBS was confirmed by analyzing its correlation with characteristics similar to those of the tool of this study, such as digital addiction and academic burnout. The results indicated a significant positive correlation. Thus, universities should provide education and support regarding academic burnout in the digital environment and ensure that students have access to appropriate support at all times. In a sustainable digital learning environment, students can overcome academic burnout and develop healthy study habits.

This study had three aims. The first was to evaluate the Korean version of the DBS, which comprises 24 items divided into 3 sub-factors, through the rigorous translation of the tool and verification of its validity and reliability. The Korean version of the DBS was theoretically and statistically proven to have high validity and reliability. The second aim was to contribute to understanding the concept of digital burnout through factor analysis. This clarification may contribute to the development of early detection and intervention programs in educational and clinical settings. The third aim was to measure individual levels of digital burnout in the era of digitalization, allowing for careful observation and awareness of university students’ digital usage patterns.

Students are constantly exposed to various digital devices, and without realizing it, may experience digital burnout from spending too much time thereon. However, these individuals may be unaware of their situation, making it challenging to find solutions. Early signs of mental and physical fatigue can be detected by identifying frequently used digital platforms, usage times, and emotional states, emphasizing the importance of assessing digital aging, digital deprivation, and emotional exhaustion. Following this study, subsequent diagnostic research is required to accurately evaluate and distinguish digital burnout among university students. Through such research, the level of risk of digital burnout can be accurately assessed and appropriate support and intervention provided.

### 5.1 Implications

Based on the results of this study, it is recommended that the characteristics of individuals based on sub-factors be identified to propose diverse and personalized strategies to promote healthy digital usage education. A program could be developed that encourages regular breaks from digital devices, which especially limits screen time before sleeping and promotes physical and mental rest through offline activities such as exercise. In addition, enhancing digital control through digital addiction programs and effective time management education can reduce digital deprivation. Finally, incorporating leisure activities such as meditation and yoga can alleviate emotional exhaustion and contribute to overall health and an improved quality of life.

## 6 Conclusion

The Korean version of the DBS has good reliability and validity and can be used as a tool to assess the level of risk of digital burnout and provide appropriate support and intervention.

## 7 Limitations

Since data for the Korean version of the DBS were collected online, possibly, only individuals with high Internet accessibility participated. Moreover, the study focused on Generation Z university students, who are characterized by high digital usage, which limits the representativeness of the sample and generalizability of the results. In future, several considerations are necessary. First, an analysis of different age groups such as working professionals, middle-aged individuals, and the older adult is needed to reconfirm the factor structure of digital burnout based on age and occupational group. Second, using self-reported survey methods may lead to overestimation or underestimation of one’s behavior or habits. Therefore, in future research, the incorporation of objective data collection techniques such as vitality indicators or stress measurement technologies is essential. Third, this study employed a cross-sectional design, collecting data at a single point in time, which limited the ability to track the dynamic nature of digital burnout. Future research should employ a longitudinal study design to establish the causal relationship between digital usage and burnout, and to identify factors that trigger digital burnout.

## Data availability statement

The original contributions presented in the study are included in the article/supplementary material, further inquiries can be directed to the corresponding author.

## Author contributions

S-YC: Conceptualization, Formal analysis, Methodology, Writing – original draft, Writing – review & editing. J-HK: Writing – original draft, Writing – review & editing, Conceptualization, Funding acquisition, Methodology.
